# Structure characterization of novel heteropolysaccharides from *Pteridium revolutum* with antioxidant and antiglycated activities

**DOI:** 10.1016/j.fochx.2023.100826

**Published:** 2023-08-05

**Authors:** Kui-Wu Wang, Xin-Yuan Sheng, Bin Wu, Hong Wang, Jian-Bo Chen, Shi-Wei Wang

**Affiliations:** a School of Food Science and Biotechnology, Zhejiang Gongshang University, Hangzhou 310018, China; b Ocean College, Zhejiang University, Hangzhou 310058, China; c School of Pharmaceutical Science, Zhejiang University of Technology, Hangzhou 310014, China; d Medical College, Jinhua Polytechnic, No. 1118 Wuzhou Road, Jinhua 321000, China

**Keywords:** *Pteridium revolutum*, Pteridaceae, Polysaccharide, Structure characterization, Antioxidant activity, Antiglycated activity

## Abstract

This study aims to analysis the structures of polysaccharides isolated from *Pteridium revolutum* and their antioxidant and antiglycated activities. Three novel water-soluble heteropolysaccharides, named PRP0, PRP1, and PRP2, were isolated from *P. revolutum*. The average molecular weight was determined by high performance gel permeation chromatography analysis as 1.04 × 10^6^, 8.39 × 10^5^, and 7.37 × 10^5^ Da, respectively. Their structures were characterized using physicochemical and spectroscopic methods. The antioxidant and antiglycated activities were assayed *in vitro*. PRP0, PRP1, and PRP2 consist of *l*-Ara, *l*-Rha, *d*-Man, *d*-Xyl, *d*-Fuc, *d*-Gal, and *d*-Glc in different proportions. PRP1 mainly has a backbone of (1 → 3,6)-linked *d*-Man and (1 → 3)-linked *d*-Gal on main chain. PRP2 is mainly composed of (1 → 2,4)-linked *d*-Man and (1 → 3)-linked *d*-Gal on main chain. All polysaccharides have strong scavenging power on 2,2-difenil-1-picril-hidrazil and hydroxyl radicals and significantly antiglycated activity in Bovine serum albumin-Glucose model, which showing that the polysaccharides have potential application value on the functional food.

## Highlights

• Novel water-soluble polysaccharides, PRP0, PRP1, and PRP2 were isolated from *Pteridium revolutum*.
• Molecular weight and monosaccharide composition of three polysaccharides were analyzed.
• The structures of PRP1 and PRP2 were systematically characterized by a combination of chemical and spectra methods.
• PRP1 and PRP2 are highly branching polysaccharides with a backbone of *α*-galactose and *α*-mannose units.
• All polysaccharides showed significant antioxidant and antiglycated activity.

## 1 Introduction

Bracken ferns have more than 12,000 species widely distributed in uplands and marginal areas throughout Asia, Australia, Europe, North and South America, with about 2600 species in China (Kardong et al., 2013, Xu et al., 2009). Certain ferns species (*Pteridium*) are used as food or plant medicine to treat ailments in several countries (Chen et al., 2015, Wang and Wu, 2013). Many chemical compositions such as flavonoids, terpenoids, and steroids with various bioactivities including antioxidant, antibacterial, anti-Alzheimer’s disease, antiosteoporosis, hypolipidemic and hypoglycemic activities have been isolated from *Pteridium* genus (Castillo et al., 2003, Song et al., 2017, Zhao et al., 2022). *Pteridium revolutum* (Blume) Nakai (Pteridiaceae family), widely distributed in south of Asia and north of Australia, has been used as food and ethnic medicine in traditional Chinese medicine. It has the functions of removing dampness, diuresis, antipyretic and insect repellent.

Plant polysaccharides have drawn considerable attention from the world because of their potential application value in various industries (Sun et al., 2008). Previous works have demonstrated that polysaccharides possess essential pharmacological activities including antioxidant, antiglycated, anti-inflammatory, hypolipidemic, antimicrobial and immunoregulatory (Chen et al., 2019, Chen et al., 2023, Long et al., 2022, Song et al., 2019, Wang et al., 2022, Zhu et al., 2019). Polysaccharides isolated from the Pteridaceae family were also reported have antioxidant and immunomodulatory activity (Xu et al., 2009, Song et al., 2017, Zhao et al., 2022). So far, the detailed structure of polysaccharides from *P. revolutum* still unclear. Therefore, the present work aimed to investigate the extraction, separation, structure characterization and the bioactivity of polysaccharides from this plant. The results exhibit that the novel polysaccharides have strong antioxidant and antiglycated activities in a concentration dependent manner. It could be used as a functional ingredient in food to reduce the formation of glycosylation products of protein.

## 2 Materials and methods

### 2.1 Polysaccharides preparation from *P. revolutum*

The sample of *P. revolutum* was collected from Suichang County, Zhejiang province, China. A voucher specimen was deposited in Zhejiang Gongshang University, Hangzhou, China. The dried aerial part of *P. revolutum* was ground to pass a 60-mesh sieve and extracted by refluxing ethanol (95% v/v, 2 × 2 h) to remove liposoluble compounds. Then, the sample was extracted with hot distilled water (10 vol, 90 °C, 3 × 3 h). The extracts obtained were vacuum concentrated at 55 °C by a rotary evaporator (Buchi R-210, BUCHI Labortechnik AG, Switzerland) and precipitated with ethanol (75% v/v, 4 °C, 24 h). The sample were centrifuged (8,000 g, 5 min). The precipitate was re-dissolved in ultra-pure water to remove the protein using the Sevage method (*n*-butanol: chloroform, 1:4 v/v). The aqueous phase was concentrated, dialyzed (3,500 molecular weight cut off (MWCO)) against distilled water for at least 2 d. Finally, the solution was vacuum concentrated (55 °C) and lyophilized to offer the crude polysaccharide (cPRP).

The cPRP sample was re-dissolved in ultra-pure water, filtered (0.45 μm, Millipore, USA) and chromatographed using DEAE Sepharose Fast Flow (DSFF, GE Healthcare, USA) chromatography column (CC, 5.0 cm × 50 cm), eluted with ultra-pure water and the NaCl (0.1 ∼ 0.5 M) successively. The eluent (10 mL/tube) was checked using the phenol–sulphuric acid method and then pooled, dialyzed, and lyophilized. The fractions eluted by ultra-pure water, 0.1 and 0.2 M NaCl were further purified on a Sephadex G-200 gel (GE Healthcare, USA) CC (5.0 cm × 50 cm), eluted with ultra-pure water to obtain three polysaccharides PRP0, PRP1, and PRP2 (Song et al., 2017).

### 2.2 General analysis of purified polysaccharides

The molecular weight (MW) and homogeneity analysis of the three purified polysaccharides were achieved by high performance gel permeation chromatography (HPGPC, Waters 2695, Evaporative light scattering detector (ELSD) 2424, Waters Corporation. USA) using a Ultra- hydrogel 2000 column (7.8 × 300 mm), eluted by ultra-pure water (0.30 mL/min). The average MW was determined using the calibration curve, which was prepared with standards *T*-series Dextran (T-670, 270, 150, 50, 10) (Song et al., 2017).

The monosaccharide compositions of PRP0, PRP1, and PRP2 were analyzed according to the method reported before (Song et al., 2017). Briefly, polysaccharide (5 mg) was hydrolyzed using trifluoroacetic acid (TFA, 2.0 M, 2.0 mL, 120 °C, 3 h). The hydrolysate was co-concentrated three times with methanol and then reduced with pyridine (1 mL) and hydroxylamine hydrochloride (5 mL, 90 °C, 30 min), acetylation by acetic anhydride (1 mL, 90 °C, 60 min). The monosaccharides of standards [*l*-arabinose (*l*-Ara), *l*-rhamnose (*l*-Rha), *d*-mannose (*d*-Man), *d*-xylose (*d*-Xyl), *d*-fucose (*d*-Fuc), *d*-galactose (*d*-Gal), and *d*-glucose (*d*-Glc)] and inositol were acetylated with the same method. All the resulting aldononitrile acetates were detected by Gas Chromatography (GC, Agilent 7890A, DB-5 MS column, hydrogen flame ionization detector, Agilent Technologies Inc., USA) using a temperature program of 120 °C (maintain 3 min), raised to 210 °C (3 °C/min, maintain 15 min). Carrier gas: N_2_, 1.0 mL/min. Injector/Detector temperature: 250/280 °C.

### 2.3 Partial acid hydrolysis

The purified polysaccharides (each 10 mg) were hydrolyzed by TFA (2.0 M, 100 °C, 2 h) (Song et al., 2017). The product was dialyzed (MWCO 3,500) in ultra-pure water for 2 d. The dialyzable fraction (outside of the bag, O) and the non-dialyzable fraction (inside of the bag, I) were lyophilized separately, offering the partially hydrolyzed samples PRP0-O, PRP1-O, PRP2-O, PRP0-I, PRP1-I, and PRP2-I, respectively. Monosaccharide compositions of these six samples were tested by GC as *Section 2.2*.

### 2.4 Periodate oxidation and Smith degradation

Twenty milligrams of PRP0, PRP1, or PRP2 sample were oxidized with NaIO_4_ (15 mM, 25 mL, 120 h), respectively. Ethylene glycol (1 mL) was used to destroy the excess NaIO_4_. The consumption of periodate was detected by Ultraviolet spectroscopy (UV, 223 nm). The generated products of formic acid were titrated by NaOH (0.01 M). After reduced by NaBH_4_ and neutralized with HOAc to pH 7.0, the solution was then hydrolyzed in TFA (2 M, 120 °C, 3 h) and analyzed the monosaccharide compositions by GC (Zhao et al., 2022).

### 2.5 Methylation analysis

The samples of PRP1 and PRP2 (each 20.0 mg) were methylated with the method reported (Song et al., 2017) and detected by Infrared spectroscopy (IR). The methylated samples were hydrolyzed, reduced and acetylated, successively. The obtained alditol acetates were detected by Gas chromatography-mass spectrometry (GC–MS). PRP0 did not methylate because of no more sample. GC–MS analysis: Agilent 5975C system (Agilent Technologies Inc., USA), DB-17 MS column. The starting temperature of the column is 50 °C, then raised to 230 °C (4 °C/min), finally reached to 280 °C (10 °C/min, maintain 15 min). Ion-source: 230 °C. N_2_: 1.0 mL/min.

### 2.6 UV, IR and NMR spectroscopy

UV absorption spectrum (200–400 nm) was detected on a Shimadzu UV-1880 spectrophotometer (Shimadzu Corporation, Japan). IR spectrum (4000–400 cm^−1^) was tested using a Nicolet iS 10 Spectrometer (Thermo Scientific, USA). 1D and 2D-NMR (Nuclear Magnetic Resonance) spectra were recorded with a Bruker AVANCE III 500 spectrometer (Bruker Corporation, Germany) at 60 °C (333 K) dissolved in D_2_O (99.9%).

### 2.7 Antioxidant activities

The scavenging capacity of four polysaccharides (cPRP, PRP0, PRP1 and PRP2) on 2,2-difenil-1-picril-hidrazil (DPPH) and hydroxyl radicals (•OH) was evaluated according to previous methods (Wang et al., 2021).

### 2.8 Antiglycated activity

#### 2.8.1 BSA-Glucose glycation model

The anti-glycation ability of PRPs was assayed using Bovine serum albumin-Glucose (BSA-Glu) model according to Hafsa’s method (Hafsa et al., 2016) with some modifications. The total 5.0 mL of reaction mixture consisted of phosphate buffer (pH 7.0, 0.1 M, 0.2% NaN_3_), BSA (10 mg/mL), glucose (0.5 M), and samples with different concentrations (0.25 ∼ 2.0 mg/mL). Negative control: no aminoguanidine (AG) and polysaccharide. Positive control: AG instead of polysaccharide. All the mixtures were reacted in dark (100 °C, 40 min). The mixture is cooled in ice water bath and stored at 4 ℃ for the following experiments. The fluorescence of the solution was measured using at an excitation/emission wavelength of *λ*_380_/*λ*_450_ nm (LS55 spectrofluorometer, Perkin-Elmer, USA). The inhibition activity was calculated as following equation (Eq).

$$Inhibition\;\left(\%\right)=[1-\left(F_{1}/F_{0}\right)]\times 100$$

*F*_0_: Fluorescence intensity of negative control. *F*_1_: Fluorescence intensity of sample/AG solution.

#### 2.8.2 Analysis of amadori products

The amadori products were determined by method reported before (Zhu et al., 2019). 0.1 mL glycated BSA was added to 0.1 mL 0.15 mM NBT reagent (Dissolved in 100 mM sodium carbonate buffer, pH 10.35). The mixture was incubated for 30 mins at room temperature and the absorbance was tested at *λ*_530_ nm on a Syhergy H1 Microplate Reader (BioTek, USA).

#### 2.8.3 Analysis of dicarbonyl compounds

The content of dicarbonyl compounds were measured by Girard-T assay (Zhu et al., 2019). 0.4 mL glycated BSA was added into the mixed solution containing Girard-T stock solution (0.2 mL, 500 mM) and sodium formate (3.4 mL, 500 mM, pH 2.9) and then reacted for 1 h at room temperature. The absorbance was determined at *λ*_294_ nm (UV-1880 spectrophotometer, Shimadzu Corporation, Japan). Glyoxal was used a standard to make a calibration curve.

#### 2.8.4 Analysis of pentosidine

The content of pentosidine was determined by a LS55 spectrofluorometer (Sun et al., 2010). The fluorescence of the glycated solution was determined at an excitation/emission wavelength of *λ*_335_/*λ*_385_ nm. The effect on pentosidine was calculated as following Eq.

$$Inhibition\;\left(\%\right)=[1-\left(F_{1}/F_{0}\right)]\times 100$$

*F*_0_: Fluorescence intensity of negative control. *F*_1_: Fluorescence intensity of sample/AG solution.

#### 2.8.5 Determination of protein carbonyl content

The level of protein carbonyl group (PCO) was evaluated using reported method (Hafsa et al., 2016). Briefly, 0.2 mL of glycated BSA solution was reacted with 1.0 mL of 2, 4-dinitrophenyl- hydrazine (DNPH, 10 mM) in 2.0 M HCl at room temperature for 1 h in the dark (Mix with a vortex mixer every 10 min). 1.0 mL trichloroacetic acid (20%, w/v) was added to precipitate the protein and then centrifuged (8,000 g, 10 min, 4 °C). The precipitate was washed 3 times with 2.0 mL of ethanol/ethyl acetate solution (1:1, v/v), then the final protein was re-suspended in guanidine hydrochloride (2.0 mL, 6.0 M, pH 2.3). The absorbance was recorded at *λ*_370_ nm. The PCO content (nmol carbonyl/mg protein) was calculated using coefficient (*ε* = 22,000 M^−1^ cm^−1^).

#### 2.8.6 Determination of thiol group

The content of thiol group was measured by Ellman method (Hafsa et al., 2016). 1.0 mL of reaction solution included 0.2 mL of glycated BSA, 0.3 mL Tris buffer (0.2 M, pH 8.2), 20 μL of Ellman reagent (0.1 mM 5,5′-Dithiobis-(2-nitrobenzoic acid), DTNB) and 0.48 mL of methanol. After reacted for 15 min at room temperature, the mixture was centrifuged at 8,000 g for 10 min. The absorbance was recorded at *λ*_412_ nm. The content of thiol group (nmol thiol group/mg protein) was calculated using extinction coefficient of the DTNB (ε = 13,600 M^−1^ cm^−1^).

### 2.9 Statistical analysis

Statistical analysis was analyzed by T-test to assess the significance (SPSS 20.0) of difference between groups and all data were given as mean ± SD.

## 3 Results and discussion

### 3.1 Preparation, molecular weight and monosaccharide composition determination

**Fig. 1:**
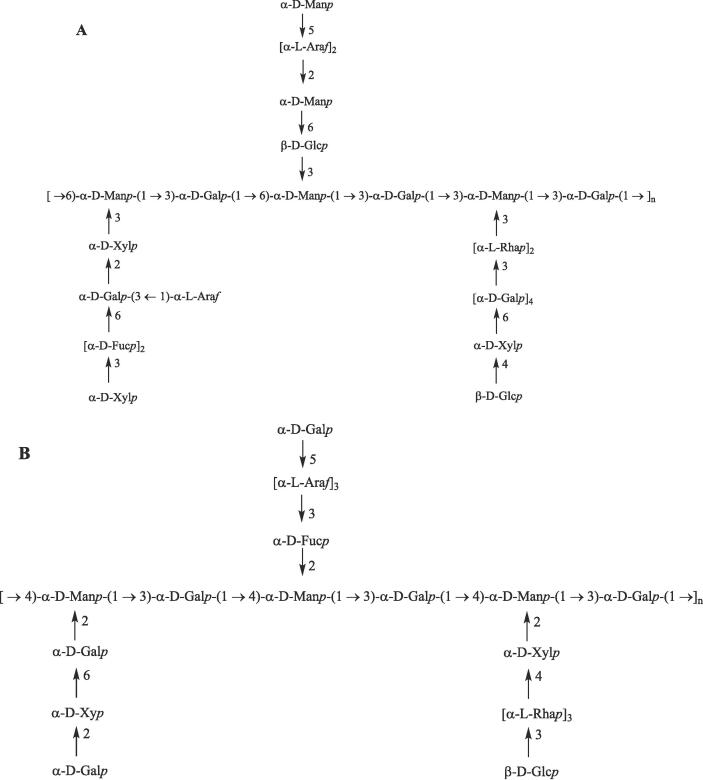
Possible structures of PRP1 (A) and PRP2 (B).

**Fig. 2:**
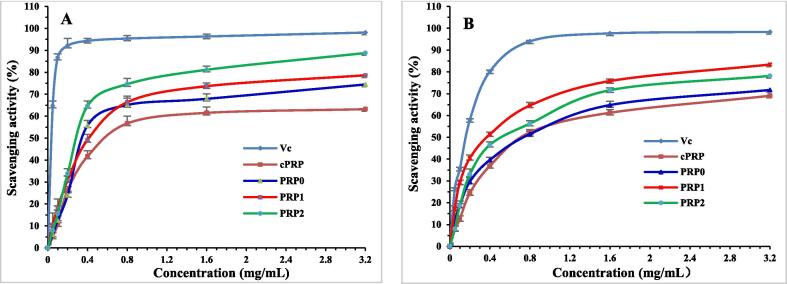
Antioxidant activities of polysaccharides from *P. revolutum*. Scavenging activity of DPPH (A), •OH (B) (n = 3).

cPRP was obtained from the *P. revolutum* by hot distilled water extraction, precipitated in 75% EtOH (v/v), deproteinated and freeze-dried. cPRP gave four peaks on the DSFF CC for purification (S-Fig. 1). The three major fractions, eluted with ultra-pure water, 0.1 and 0.2 M NaCl solution, were further chromatographed by Sephadex G-200 (S-Fig. 2) to provide three purified novel polysaccharides (named PRP0, PRP1, and PRP2).

**Fig. 3:**
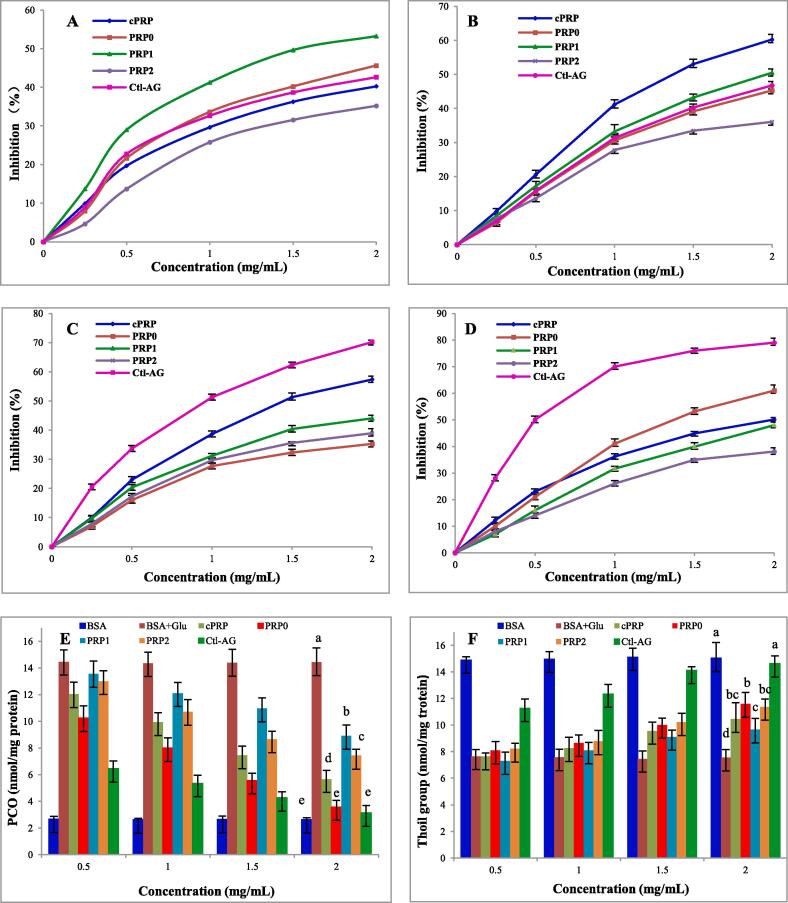
Antiglycation capability in the BSA-Glu models. Inhibit amadori prudocts formation; (B) Inhibit dicarbony compounds formation; (C) Inhibit pentosidine formation; (D) Inhibit AGEs formation; (E) The effect of on PCO formation; (F) The effect of on protein thiol groups formation. AG: Aminoguanidine. P < 0.01.

The homogeneity and average molecular weight of three novel polysaccharides was identified by HPGPC (S-Fig. 3). The average MW was 1.04 × 10^6^, 8.39 × 10^5^, and 7.37 × 10^5^ Da, which calculated using the Standard curve of Dextran molecular weight (S-Fig. 4), respectively. As showed in UV spectra (S-Fig. 5), three polysaccharides were free of protein because of the weak absorption at 260–280 nm.

The products obtained from complete hydrolysis with 2.0 M TFA of PRP0, PRP1 and PRP2 were tested by GC. The results (S-Fig. 6, Table 1) indicated the three purified polysaccharides presence of alditol acetates of arabinose, rhamnose, mannose, xylose, fucose, galactose, and glucose in the molar ratio as 8.23: 2.76: 3.61: 1.24: 1.00: 10.14: 8.52 (PRP0), 5.85: 4.18: 4.19: 2.16: 2.48: 10.26: 1.00 (PRP1) and 4.02: 4.34: 4.04: 2.81: 3.35: 9.11: 1.00 (PRP2). Galactose is the main sugar composition of the three polysaccharides. The monosaccharide compositions of these three polysaccharides are different from reported polysaccharides obtained from *Pteridium* plants. Thus, they are novel natural polysaccharides isolated from *P. revolutum.*

**Table 1:** Results of monosaccharide composition (MC) and partial acid hydrolysis of PRPs (Molar ratio).

	Samples	Ara	Rha	Man	Xyl	Fuc	Gal	Glc
Monosaccharide composition	PRP0	8.23	2.76	3.61	1.24	1.00	10.14	8.52
	PRP1	5.85	4.18	4.19	2.16	2.48	10.26	1.00
	PRP2	4.02	4.34	4.04	2.81	3.35	9.11	1.00

Partial acid hydrolysis	PRP0-O	5.71	1.78	3.92	1.00	1.07	8.89	11.12
	PRP0-I	–	1.16	15.38	1.00	–	3.59	9.32
	PRP1-O	4.10	2.53	1.51	1.02	1.78	5.63	1.00
	PRP1-I	1.00	1.86	10.33	3.82	1.36	12.76	1.70
	PRP2-O	7.11	5.85	1.00	2.12	4.95	12.87	1.02
	PRP2-I	1.00	2.38	11.56	5.84	3.34	10.65	1.06

### 3.2 Structural characterization

The chemical structures of three purified polysaccharides were then systemically characterized by chemical and spectroscopy methods.

The three polysaccharides were partially hydrolyzed with TFA and dialyzed with MWCO 3,500 member. The monosaccharide compositions of the out and in the dialysis bag were collected and analyzed separately. The results (S-Fig. 7, Table 1) showed that PRP0-O was consisted of *l*-Ara, *l*-Rha, *d*-Man, *d*-Xyl, *d*-Fuc, *d*-Gal, and *d*-Glc in a molar proportion of 5.71: 1.78: 3.92: 1.00: 1.07: 8.89: 11.12, PRP0-I contained *l*-Rha, *d*-Man, *d*-Xyl, *d*-Fuc, and *d*-Gal of 1.16: 15.38: 1.00: 3.59: 9.32, suggesting that almost *d*-Man was present as the main chain, *l*-Ara, *d*-Fuc, and *d*-Gal were mainly present on the side chain, and *d*-Glc were both in main chain and side chain. Similarly, PRP1-O was consisted of *l*-Ara, *l*-Rha, *d*-Man, *d*-Xyl, *d*-Fuc, *d*-Gal, and *d*-Glc of 4.10: 2.53: 1.51: 1.02: 5.63: 1.00: 1.78, PRP1-I contained the same monosaccharides of 1.00: 1.86: 10.33: 3.82: 12.76: 1.70: 1.36. To PRP2-O and PRP2-I, the molar ratios were 7.11: 5.85: 1.00: 2.12: 12.87: 1.02: 4.95 (PRP2-O), and 1.00: 2.38: 11.56: 5.84: 10.65: 1.06: 3.34 (PRP2-I), respectively. These results demonstrated that almost *d*-Man was present as the main chain and *d*-Gal was both in main chain and side chain in PRP1 and PRP2. Therefore, the backbone of PRP0 was composed of *d*-Man and *d*-Glc (3:2) because these two monosaccharides were mainly found in PRP0-I after 2 M TFA hydrolysis. While the backbones of PRP1 and PRP2 were consisted of *d*-Man and *d*-Gal in 1: 1.2 and 1.1: 1, respectively.

The three-polysaccharide samples were then oxidized with NaIO_4_ (15 mM, 120 h). 0.935, 1.012, or 0.805 mol periodate was consumed per mole of sugar residue to produce 0.400, 0.364, or 0.238 mol HCOOH for PRP0, PRP1 or PRP2, respectively (S-Table 1). These data indicated that the (1 → 6)-linked and/or *T*-glycosyl (non-reducing terminal residue) bonds amounted to 40.0% in PRP0, 36.4% in PRP1, 23.8% in PRP2, with the (1 → 2)-/(1 → 4)-/(1 → 4,6)-/(1 → 2,6)-linked glycosyl bonds amounted to 13.5% (PRP0), 28.4% (PRP1), 32.9% (PRP2), and (1 → 3)-/(1 → 2,3)-/(1 → 2,4)-/(1 → 3,4)-/(1 → 3,6)-/(1 → 2,3,4)-linked glycosyl bonds amounted to 46.5% (PRP0), 35.2% (PRP1), 43.3% (PRP2), respectively. The GC–MS spectra of Smith degradation products indicated the existence of erythritol, glycerol, rhamnose, arabinose, fucose, mannose and galactose (S-Fig. 8, S-Table 2), revealing that the residues of these glycosyl bonds were (1 → 2,3)-/(1 → 2,3,4)-/(1 → 2,4)-/(1 → 3)-/(1 → 3,4)- and/or (1 → 3,6)-linked, which were unoxidizable. The absence of xylose in three polysaccharides and the absence of glucose in PRP1 and PRP2 of the oxidation products made clear that xylose and glucose were in oxidizable linkages as (1 → )-/(1 → 2)-/(1 → 2,6)-/(1 → 4)-/(1 → 4,6)- and/or (1 → 6)-linkage, which confirmed by the presence of erythritol and glycerol. The absence of arabinose suggested that the residues were (1 → 4)-linked Ara*f*.

The GC–MS analysis of methylated polysaccharides of PRP1and PRP2 showed different major peaks (S-Fig. 9). Fifteen types of residues, 2,3-Me_2_-Ara*f*, 2,3,5-Me_3_-Ara*f*, 2,4-Me_2_-Fuc*p*, 2,4-Me_2_- Gal*p*, 2,3,4-Me_3_-Gal*p*, 2,4,6-Me_3_-Gal*p*, 2,4-Me_2_-Rha*p*, 2,3,4-Me_3_-Glc*p*, 2,3,4,6-Me_4_-Glc*p*, 2,4-Me_2_- Man*p*, 3,4,6-Me_3_-Man*p*, 2,3,4,6-Me_4_-Man*p*, 2,3-Me_2_-Xyl*p*, 3,4-Me_2_-Xyl*p*, and 2,3,4-Me_3_-Xyl*p* were identified in PRP-1. While 2,3-Me_2_-Ara*p*, 2,4-Me_2_-Fuc*p*, 2,4,6-Me_3_-Gal*p*, 2,3,4,6-Me_4_-Gal*p*, 2,3,4-Me_3_-Glc*p*, 2,3,4,6-Me_4_-Glc*p*, 2,4-Me_2_-Rha*p*, 3,6-Me_2_-Man*p*, 2,3-Me_2_-Xyl*p*, and 3,4-Me_2_- Xyl*p* were detected in PRP-2 (Table 2).

**Table 2:** GC–MS data for methylation analysis of PRP1 and PRP2.

Methylated Sugars	Linkages	Major Mass Fragments (*m*/*z*)	Molar ratio	
			PRP1	PRP2
2,3,5-Me_3_-Ara*p*	1-Linked-Ara*f*	71, 87, 117, 129, 161	1.2	–
2,3-Me_2_-Ara*p*	1,5-Linked Ara*f*	71, 87, 101, 117, 129, 189	1.8	2.6
2,4-Me_2_-Fuc*p*	1,3-Linked Fuc*p*	81, 101, 117, 126, 155, 207, 233	2.3	1.2
2,3,4,6-Me_4_-Gal*p*	1-Linked Gal*p*	87, 101, 117, 129, 145, 161, 205	–	1.1
2,4,6-Me_3_-Galp	1,3-Linked Gal*p*	87, 129, 189, 207, 261, 281	2.6	2.6
2, 3, 4-Me_3_-Gal*p*	1,6-Linked Gal*p*	87, 101, 117, 129, 173, 189, 207	3.6	–
2, 4-Me_2_-Galp	1,3,6-Linked Gal*p*	87, 99, 101, 129, 149, 161, 233	1.2	–
2,3,4,6-Me_4_-Glc*p*	1-Linked Glc*p*	71, 87, 101, 117, 129, 161, 191	1.0	1.0
2,3,4-Me_3_-Glc*p*	1,6-Linked Glc*p*	87, 101, 113, 117, 129, 161, 233	1.1	1.2
2,3,4,6-Me_4_-Man*p*	1-Linked Man*p*	89, 101, 117, 131, 161, 175, 207	1.1	–
3,4,6-Me_3_-Man*p*	1,2-Linked Man*p*	71, 87, 101, 117, 129, 161, 189	1.8	–
3,6-Me_2_-Man*p*	1,2,4-Linked Man*p*	87, 111, 129, 189, 207, 233, 253, 281	–	2.7
2,4-Me_2_-Man*p*	1,3,6-Linked Man*p*	87, 99, 117, 129, 173, 189, 207, 233	2.8	–
2,4-Me_2_-Rha*p*	1,3-Linked Rha*p*	43, 101, 117, 13, 207, 233	2.1	2.6
2,3,4-Me_3_-Xyl*p*	1-Linked-Xyl*p*	71, 87, 101, 117, 129, 161	1.1	–
3,4-Me_2_-Xyl*p*	1,2-Linked Xyl*p*	87, 101, 129, 145, 161, 189	1.2	1.0
2,3-Me_2_-Xyl*p*	1,4-Linked Xyl*p*	87, 101, 117, 129, 173, 189, 207	1.0	1.1

The above results made clear that (1 → 3)-linked *d*-Glc and (1 → 3,6)-linked *d*-Man were the major residues of the main-chain in PRP1. (1 → 5)-Linked *l*-Ara, (1 → 3)-linked *l*-Rha, (1 → 2)- linked *d*-Man, (1 → 2)- and (1 → 4)-linked *d*-Xyl, (1 → 3)-linked *d*-Fuc, (1 → 6)-linked *d*-Glc, (1 → 3,6)- and (1 → 6)-linked *d*-Gal joined to position C-3 of (1 → 3,6)-linked *d*-Man as branches. *l*-Ara, *l-*Rha, *d*-Xyl, and *d*-Glc were at the termini of the side-chains. PRP2 mainly consisted of a backbone of (1 → 2,4)-linked *d*-Man and (1 → 3)-linked *d*-Gal, with (1 → 5)-linked *l*-Ara, (1 → 3)-linked *l*-Rha, (1 → 2)- and (1 → 4)-linked *d*-Xyl, (1 → 3)-linked *d*-Fuc, (1 → 6)-linked *d*-Gal as branches. *d*-Glc and *d*-Gal were located at the end of side-chains.

The further chemical structures elucidation of PRP1 and PRP2 were conducted by 1D and 2D NMR spectra. The anomeric signals of all major residues of PRP1 in ^1^H (S-Fig. 10A) and ^13^C NMR spectrum (S-Fig. 10B) were assigned by comparing with reported data and the 2D NMR information (S-Fig. 10C-F). Fifteen anomeric signals from δ_C_ 98 to 112 ppm (Table 3) were identified of PRP1. The signal at δ_C_ 101.70 ppm was were attributed to the (1 → 6)-linked *d-*Gal*p* residue, the corresponding anomeric proton signal appeared at δ_H_ 5.02 ppm, which affirmed the (1 → 6)-linked *d*-Gal*p* was *α*-*d*-conformation (Cui et al., 2007, Pramanik et al., 2005) (Table 3). Similarly, the signals at δ_H_/δ_C_ 5.39/98.65 and 4.82/101.88 ppm belonged to H1/C1 of (1 → 3)- and (1 → 3,6)-linked *α-d*-Gal*p* residues, respectively (Jing et al., 2014, Li et al., 2021, Siwińska et al., 2020). The signals of H1/C1 at δ_H_/δ_C_ 5.86/111.19, 5.86/110.78, 5.57/98.50, 5.22/100.55, 5.18/101.18, 5.17/101.34, 5.04/101.53, 5.00/102.64, 4.90/102.20, 4.88/102.43, 4.52/103.12, and 4.40/102.83 confirmed the presence of T-*α-l*-Ara*f*-(1→ (Zhang et al., 2021a), →5)-*α-l*-Ara*f*-(1→ (Makarova and Shakhmatov, 2020, Chen et al., 2019), →3,6)-*α-d*-Man*p*-(1→ (Chen et al., 2019, Roslund et al., 2011), →2)-*α-d*-Man*p*-(1→ (Smiderle et al., 2013, Naumenko et al., 2020), →4)-*α-d*-Xyl*p*-(1→ (He et al., 2013), →2)-*α-d*-Xyl*p*-(1→ (Makarova & Shakhmatov, 2021), T-*α-d*-Man*p*-(1→ (Roslund et al., 2011, Liu et al., 2021a), →3)-*α-L*- Rha*p*-(1→ (Bellich et al., 2021), T-*α-d*-Xyl*p*-(1→ (Makarova & Shakhmatov, 2021), →3)-*α-D*-Fuc*p*-(1→ (Liu et al., 2021b, Szpakowska et al., 2020), →6)-*β-d*-Glc*p*- (1→ (Liu et al., 2021b, Li et al., 2020), and T-*β-d*-Glc*p*-(1→ residues (Li et al., 2020, Wang and Guo, 2020), respectively. The NMR signals in the upfield at *δ*_H_/*δ*_C_ 1.15–1.25/16.5–18.5 ppm were assigned to –CH_3_ groups, confirming the presence of fucose and/or rhamnose.

**Table 3:** The NMR chemical shift assignments of PRP1 and PRP2 (^1^H, 500 MHz, ^13^C, 125 MHz, D_2_O, *δ* in ppm).

	Residues	H1/C1	H2/C2	H3/C3	H4/C4	H5/C5	H6/C6
PRP1	T-*α-l*-Ara*f*-(1→ (A)	5.86 111.19	4.11 81.06	3.93 76.94	4.40 80.95	3.68 62.42	-- --
	→5)-*α-l*-Ara*f*-(1→ (B)	5.86 110.78	4.11 80.56	4.03 76.73	4.40 80.95	3.85 66.65	-- --
	→3)-*α-d*-Fuc*p*-(1→ (C)	4.88 102.43	3.89 68.83	4.00 81.06	3.85 73.70	4.24 68.46	1.16 16.70
	→3)-*α-d*-Gal*p*-(1→ (D)	5.39 98.65	3.89 68.83	4.24 79.77	4.11 69.63	4.18 68.46	3.75 60.95
	→6)-*α-d*-Gal*p*-(1→ (E)	5.02 101.70	3.85 70.74	3.59 71.55	3.85 70.74	3.93 69.98	3.85 66.74
	→3,6)-*α-d*-Gal*p*-(1→ (F)	4.82 101.88	3.85 70.35	4.06 76.94	3.93 70.35	3.68 74.53	3.85 66.74
	T-*β-d*-Glc*p*-(1→ (G)	4.40 102.83	4.02 72.87	3.82 76.72	3.22 69.63	3.35 76.25	3.59 60.89
	→6)-*β-d*-Glc*p*-(1→ (H)	4.52 103.12	4.02 72.87	3.82 76.25	3.85 69.98	3.59 74.53	3.73 66.65
	T-*α-d*-Man*p*-(1→ (I)	5.04 101.53	3.85 69.63	4.15 69.51	3.93 68.46	3.90 71.55	3.68 62.42
	→2)-*α-d*-Man*p*-(1→ (J)	5.22 100.55	4.02 76.94	3.69 70.35	4.18 69.51	3.60 74.53	3.68 62.76
	→3,6)-*α-d*-Man*p*-(1→ (K)	5.57 98.15	4.04 72.49	3.59 81.06	3.35 74.53	3.68 73.70	3.85 66.57
	→3)-*α-l*-Rha*p*-(1→ (L)	5.00 102.64	4.24 69.63	3.85 79.07	3.59 72.49	3.80 69.98	1.24 18.39
	T-*α-d*-Xyl*p*-(1→ (M)	4.90 102.20	3.60 74.53	3.73 76.94	3.59 72.87	3.68 63.01	-- --
	→2)-*α-d*-Xyl*p*-(1→ (N)	5.17 101.34	3.73 80.95	3.82 74.53	3.68 72.15	3.68 63.01	-- --
	→4)-*α-d*-Xyl*p*-(1→ (O)	5.18 101.18	3.59 72.87	3.34 70.74	3.73 76.93	3.68 63.01	-- --

PRP2	→5)-*α-l*-Ara*f*-(1→ (A)	5.88 110.82	4.12 80.45	4.00 76.94	4.39 80.58	3.93 66.63	-- --
	→3)-*α-d*-Fuc*p*-(1→ (B)	4.89 101.86	3.93 69.23	4.09 80.58	3.93 73.45	4.39 68.49	1.16 16.70
	T-*α-d*-Gal*p*-(1→ (C)	4.92 98.66	3.93 69.79	3.85 70.36	3.93 72.85	4.09 69.92	3.63, 3.93 62.46
	→3)*-α-d*-Gal*p*-(1→ (D)	5.40 97.85	3.95 68.59	4.28 79.82	4.20 69.50	4.21 68.49	3.63, 3.93 62.46
	→6)*-α-d*-Gal*p*-(1→ (E)	5.01 101.09	3.94 70.36	3.65 71.63	3.88 69.92	3.93 69.79	3.61, 3.93 66.67
	T-*β-d*-Glc*p*-(1→ (F)	4.39 102.43	3.94 72.85	3.63 76.94	3.22 69.79	3.65 74.54	3.63, 3.93 62.57
	→2,4)-*α-d*-Man*p*-(1→ (G)	5.06 101.39	4.39 79.10	3.60 69.50	4.56 80.22	3.65 74.54	3.63, 3.93 62.76
	→3)-*α-l*-Rha*p*-(1→ (H)	5.02 102.65	4.24 69.52	3.83 80.11	3.65 72.15	3.85 69.92	1.21 18.38
	→2)-*α-d*-Xyl*p*-(1→ (I)	5.17 101.75	3.75 80.58	3.92 74.54	3.65 72.15	3.63, 3.93 62.58	-- --
	→4)-*α-d*-Xyl*p*-(1→ (J)	5.17 101.99	3.60 72.50	3.35 69.50	3.72 76.94	3.63, 3.93 62.76	-- --

Additionally, the substitution of H atom of OH on the sugar ring results in the chemical shift of corresponding C atom downfield shifting (Roslund et al., 2011, Yao et al., 2021). PRP1 showed significant difference (about 4 ppm downfield) in the chemical shift values of C5 between →5)-*α*- *l*-Ara*f*-(1→ and T-*α*-*l*-Ara*f*-(1 →. Similarly, the ^13^C NMR absorption peak of C6 in →6)-*β-D*- Glc*p*-(1→ was downfield shifted to 66.65 ppm from 60.89 ppm in T-*β-d*-Glc*p*-(1→, C2 in →2)-*α- d*-Man*p*-(1→ was downfield shifted to 76.94 ppm from 69.63 in T-*α-d*-Man*p*-(1→, C2/C4 in →2)-*α-d*-Xyl*p*-(1→/→4)-*α-d*-Xyl*p*-(1→ were downfield shifted to 80.95/76.93 ppm from 74.53/72.87 ppm in →4)-*α-d*-Xyl*p*-(1→ (S-Fig. 10B). The chemical shift values of C3 (81.06/79.77/79.07 ppm) in →3)-*α-d*-Fuc*p*-(1→, →3)-*α-d*-Gal*p*-(1→, →3)-*α-l*-Rha*p*-(1→, C6 (66.74 ppm) in →6)-*α-D*-Gal*p*-(1→, C3/C6 (76.94/66.74 ppm) in →3,6)-*α-d*-Gal*p*-(1→, C3/C6 (81.06/66.57 ppm) in →3, 6)-*α-d*-Man*p*-(1→, were also moved to the lower field. These NMR data confirmed the glycosidic bond positions. The NMR data of the fifteen main residues were assigned and listed in Table 3.

From the NMR spectra of PRP2 (S-Fig. 11), ten anomeric H and C atoms appeared at δ_H_/δ_C_ 5.88/110.82, 5.40/97.85, 5.17/101.99, 5.17/101.75, 5.06/101.39, 5.02/102.65, 5.01/101.09, 4.92/98.66, 4.89/101.86, and 4.39/102.43 ppm (Table 3) were assigned to →5)-*α-l*-Ara*f*-(1→ (Makarova and Shakhmatov, 2020, Chen et al., 2019), →3)-*α-d*-Gal*p*-(1→ (Jing et al., 2014, Li et al., 2021, Siwińska et al., 2020), →4)-*α-d*-Xyl*p*-(1→ (He et al., 2013), →2)-*α-d*-Xyl*p*- (1→ (Makarova, & Shakhmatov, 2021), →2,4)-*α-d*-Man*p*-(1→ (Roslund et al., 2011, Song et al., 2019), →3)-*α-l*-Rha*p*-(1→ (Bellich et al., 2021), →6)-*α-d*-Gal*p*-(1→ (Cui et al., 2007, Pramanik et al., 2005), T-*α-d*-Gal*p*-(1→ (Zhang et al., 2021b), →3)-*α-d*-Fuc*p*-(1→ (Liu et al., 2021b, Szpakowska et al., 2020), and T-*β-d*-Glc*p*-(1→ (Li et al., 2020, Wang and Guo, 2020), respectively. The signals in the upfield at *δ*_H_/*δ*_C_ 1.15–1.25/16.5–18.5 ppm confirmed the presence of fucose and rhamnose.

Like PRP1, the 2-*O*-substituted and 4-*O*-substituted *α-d*-Xyl*p* units of PRP2 were confirmed by downfield C2/C4 chemical shift values at δ_C_ 80.58/76.94 ppm. The 3-*O*-substituted units of →3)-*α-l*-Rha*p*-(1→, →3)-*α-d*-Gal*p*-(1→, and →3)-*α-l*-Rha*p*-(1→ were confirmed by the down-field of C3 chemical shift values at δ_C_ 80.58/79.82/80.11 ppm, respectively. The chemical shift values of C5 (66.63 ppm) in →5)-*α-l*-Ara*f* and C6 (66.67 ppm) in →6)-*α-d*-Gal*p*-(1→ units were also downfield about 4–5 ppm. Signals relative to the →2,4)-*α-d*-Man*p*-(1→ were observed at δ_C_ 79.10 (C2) and 80.22 (C4) ppm. All the linkage positions of the desired glycosidic bonds are verified by the chemical shifts moved to lower field of the corresponding carbon atoms on the sugar ring. The NMR data of the ten main residues were listed in Table 3.

By comprehensive analyses of the information of partial acid hydrolysis, periodate oxidation, methylation, and NMR spectra, the predicted structures of two novel polysaccharides PRP1 and PRP2 are shown in Fig. 1.

### 3.3 Antioxidant activities of PRPs

#### 3.3.1 Scavenging activity on DPPH

The scavenging activity of four polysaccharides on DPPH free radicals were shown in Fig. 2A. The polysaccharides showed significant effects against DPPH free radicals. PRP0, PRP1, and PRP2 have better activity than cPRP. PRP2 has the best scavenging activity increased from 7.94 ± 1.87% to 86.92 ± 1.62% following the concentration increased from 0.05 to 3.2 mg/mL in a concentration- dependent manner. As a control, Vc showed higher activity than all polysaccharides, reaching 98.11 ± 1.09% at 3.2 mg/mL.

The scavenging activity on DPPH free radicals of PRP2 is better than reported polysaccharides PLP and PAP-3 from *P. aquilinum* (Xu et al., 2009, Zhao et al., 2022). The activity of all the isolated polysaccharides was lower than that of the oligosaccharides derived from *P. aquilinum* reported by Wang (Wang & Wu, 2013). The activity of PAP-3 is slightly better than that of PLP. The molecular weight may be an important factor antioxidant affecting activity.

#### 3.3.2 Scavenging ability on •OH

The scavenging activity of four polysaccharides on •OH were shown in Fig. 2B. The ability was enhanced according the concentration increased from 0.05 to 3.2 mg/mL. The scavenging activities of four polysaccharides could be ordered as PRP1 > PRP2 > PRP0 > cPRP, and PRP1 displayed remarkable scavenging rate of 83.37 ± 0.98% at a concentration of 3.2 mg/mL, while Vc showed higher scavenging rate of 98.34 ± 0.21% at the same concentration.

### 3.4 Antiglycation capability of PRPs in the BSA-Glu model

The inhibitory activity of PRPs on glycation was assayed using BSA-Glu model. In the early stage of glycation, the carbonyl group of glucose react with compounds containing free amino groups to form unstable Schiff bases through carbonyl amine condensation, which undergo irreversible rearrangement to form more stable Amadori products. Amadori products can react with NBT reagent to produce a colored substances, which can be measured at *λ*_530_ nm. The inhibition rate on Amadori products iomproved gradually with increasing concentration of PRPs and the positive AG (Fig. 3A). Among the tested inhibitors, the inhibitory activity was in the order of PRP1 > PRP0 > AG > cPRP > PRP2. At the concentration of 2.0 mg/mL, the inhibition rate of cPRP, PRP0, PRP1, PRP2 was 40.26 ± 1.72%, 45.66 ± 1.18%, 53.26 ± 0.59%, and 35.22 ± 1.03%, respectively.

In the intermediate stage, the dicarbonyl compounds were formed from Amadori products after oxidation and dehydration. In this research, PRPs showed inhibition activity on the formation of dicarbonyl compounds, and the inhibition rate gradually increased with the concentration increasing from 0.25 to 2.0 mg/mL. Among them, cPRP and PRP1 have better inhibitory activity than positive control AG, and cPRP exhibited the best inhibition ability with inhibition rate 60.28 ± 1.48% at 2.0 mg/mL (Fig. 3B).

In the last phase, the polymerization of carbonyl compounds with amino proteins to form advanced glycation end products (AGEs), and pentosidine is a representative compound of fluorescent AGEs. As shown in Fig. 3C and 3D, all PRPs had strong inhibitory effect on the formation of pentosidine and fluorescent AGEs. The ability of the samples to inhibit pentosidine was in the order of AG > cPRP > PRP1 > PRP2 > PRP0, while to fluorescent AGEs formation was in the order of AG > PRP0 > cPRP > PRP1 > PRP2.

The contents of PCO and free thiol group were used to evaluate the protein oxidation induced by AGEs. As shown in Fig. 3E, the carbonyl content of glycated BSA was 14.43 nmol carbonyl/mg proteins. The level of PCO decreases significantly with the PRP sample is added to glucose-glycated BSA. At the concentration of 2.0 mg/mL, cPRP, PRP0, PRP1, PRP2 displayed 5.66 ± 0.66, 3.56 ± 0.51, 8.91 ± 0.82, 7.41 ± 0.48 nmol carbonyl/mg proteins, respectively, and there was no significant difference between the activity of PRP0 and aminoguanidine (P < 0.01).

The effects of PRPs on protein thiol group level in glucose-glycated BSA were shown in Fig. 3F. The protein thiol group content was significant decrease in glycated BSA comparing to BSA solution. The addition of PRPs can increase the thiol content in glycated BSA solution. At the concentration of 2.0 mg/mL, all PRPs showed significant improvement compared to the glycated BSA, but still had a significant difference from the positive control AG (P < 0.01).

Many factors can induce the formation of AGEs and protein damage. The production of reactive oxygen species and free radical is one of the mechanisms (Shen et al., 2017, Hafsa et al., 2016). In our study, PRPs showed inhibitory activity against the formation of protein carbonyl groups and protective effect on protein thiol groups may be related to their antioxidant activities of scavenging free radicals and reactive oxygen species.

## 4 Conclusions

The preparation and structure characterization from *P. revolutum* polysaccharides were reported for the first time. The spectrometry and spectroscopy analyses indicated that PRP1 consists of (1 → 3,6)-linked *d*-Man and (1 → 3)-linked *d*-Glc, while PRP2 consists of (1 → 2,4)-linked *d*-Man and (1 → 3)-linked *d*-Gal on main chain.

Interestingly, compare the antioxidant activities between PRP1 and PRP2, PRP1 has better hydroxyl radical scavenging ability, while PRP2 has better DPPH scavenging ability. These differences may be resulted in the physiochemical and structural properties of the two polysaccharides, such as the molecular weight, monosaccharide contents, glycosidic linkage, main-chain, branched-chain, and water solubility. The difference in the content of *l*-Arabinose, *l*-Rhamnose, and *d*-Galactose may be an important factor for the activity. Moreover, polysaccharide with smaller MW usually has better performance on antioxidant activity because of better solubility. Consequently, in future research, a comprehensive understanding of the structure–activity relationships of *P. revolutum* polysaccharides will be studied.

In the BSA-Glu model, PRPs displayed inhibitory activity on the formation of Amadori products, dicarbony compounds, pentosidine, fluorescent AGEs, PCO and have a protective effect on protein thiol group. These results suggest that PRPs can be used as an natural antioxidants and the inhibitors of AGEs.

## CRediT authorship contribution statement

**Kui-Wu Wang:** Conceptualization, Methodology, Writing – review & editing, Supervision. **Xin-Yuan Sheng:** Writing – original draft, Investigation, Data curation. **Bin Wu:** Data curation, Writing – review & editing. **Hong Wang:** Writing – review & editing. **Jian-Bo Chen:** Resources. **Shi-Wei Wang:** Investigation, Validation, Data curation.

## Declaration of Competing Interest

The authors declare that they have no known competing financial interests or personal relationships that could have appeared to influence the work reported in this paper.

## Data Availability

Data will be made available on request.
