# Supplementary material for: Structure characterization of novel heteropolysaccharides from Pteridium revolutum with antioxidant and antiglycated activities
Source: Food Chem X. 2023 Aug 5;19:100826. doi: 10.1016/j.fochx.2023.100826 (PMC10534159; doi:10.1016/j.fochx.2023.100826)
Supplement: Supplementary data 1 [file mmc1.docx]

Structure characterization of novel heteropolysaccharides from *Pteridium revolutum* with antioxidant and antiglycated activities

**Kui-Wu Wang ^a, *^, Xin-Yuan Sheng ^a^, Bin Wu ^b^, Hong Wang ^c^, Jian-Bo Chen ^d^, Shi-Wei Wang ^a^**

^a^ *School of Food Science and Biotechnology, Zhejiang Gongshang University, Hangzhou 310018, China*

^b^ *Ocean College, Zhejiang University, Hangzhou 310058, China*

^c^ *School of Pharmaceutical Science, Zhejiang University of Technology, Hangzhou 310014, China*

^d^ *Medical College, Jinhua Polytechnic, No. 1118 Wuzhou Road, Jinhua 321000, China*

*****Correspondence: [wkwnpc@zjgsu.edu.cn](mailto:wkwnpc@zjgsu.edu.cn) (K.-W. Wang); Tel.: +86-571-28008975

**Supplementary Material**

**S-Fig. 1**. Purification graph of DEAE-Sepharose Fast Flow column chromatography.

**S-Fig. 2**. Purification graphs of PRP0 (A), PRP1 (B), and PRP2 (C) on Sephadex G-200 column chromatography.

**S-Fig. 3**. The HPGPC chromatograms of PRP0 (A), PRP1 (B), and PRP2 (C).

**S-Fig. 4**. Standard curve of Dextran molecular weight.

**S-Fig. 5**. The UV spectra of PRP0 (A), PRP1 (B), and PRP2 (C).

**S-Fig. 6**. GC graphs of standard monosaccharide (A), monosaccharide compositions of PRP0 (B), PRP1 (C), and PRP2 (D).

**S-Fig. 7**. GC graphs of monosaccharide compositions of PRP0-O (A), PRP0-I (B), PRP1-O (C), PRP1-I (D), PRP2-O (E), and PRP2-I (F).

**S-Fig. 8**. GC graphs of standard monosaccharide (A) and Smith degradation products of PRP0 (B), PRP1 (C), and PRP2 (D).

**S-Fig. 9**. Total ionic chromatograms of methylated PRP1 (A) and PRP2 (B).

**S-Fig. 10**. 1D and 2D NMR spectra of PRP1. (A: ^1^H NMR, B: ^13^C NMR, C: DEPT-135, D: ^1^H-^1^H COSY, E: HSQC, and F: HMBC)

**S-Fig. 11**. 1D and 2D NMR spectra of PRP2. (A: ^1^H NMR, B: ^13^C NMR, C: DEPT-135, D: ^1^H-^1^H COSY, E: HSQC, and F: HMBC)

**S-Table 1**. Results of periodic acid in oxidation of homogeneous polysaccharides

**S-Table 2**. Results of products of Smith degradation of homogeneous polysaccharides

**S-Fig. 1**. Purification graph of DEAE-Sepharose Fast Flow column chromatography.

**S-Fig. 2**. Purification graphs of PRP0 (A), PRP1 (B), and PRP2 (C) on Sephadex G-200 column chromatography.


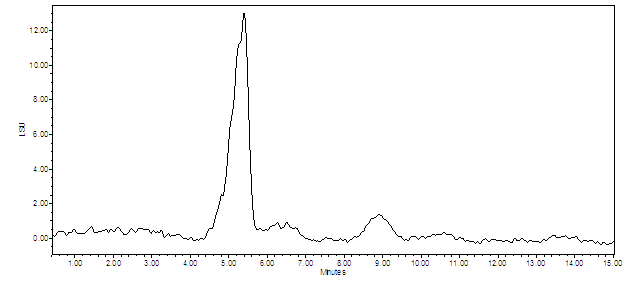

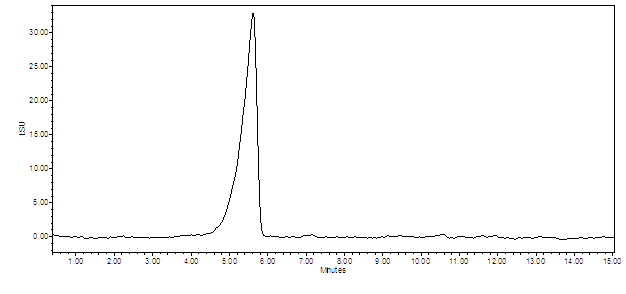

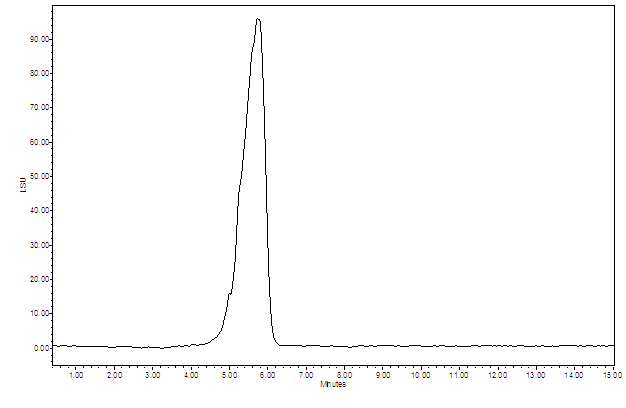


A B C

**S-Fig. 3**. The HPGPC chromatograms of PRP0 (A), PRP1 (B), and PRP2 (C).

**S-Fig. 4**. Standard curve of Dextran molecular weight.

**S-Fig. 5**. The UV spectra of PRP0 (A), PRP1 (B), and PRP2 (C).

A B

C D

S-Fig. 6. GC graphs of standard monosaccharide (A), monosaccharide compositions of PRP0 (B), PRP1 (C), and PRP2 (D).

1. (B)

(C) (D)

(E) (F)

**S-Fig. 7**. GC graphs of monosaccharide compositions of PRP0-O (A), PRP0-I (B), PRP1-O (C), PRP1-I (D), PRP2-O (E), and PRP2-I (F).


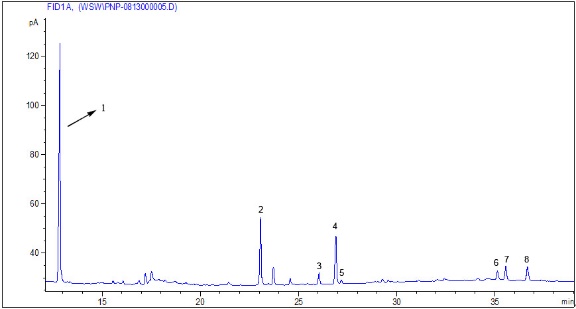


A B


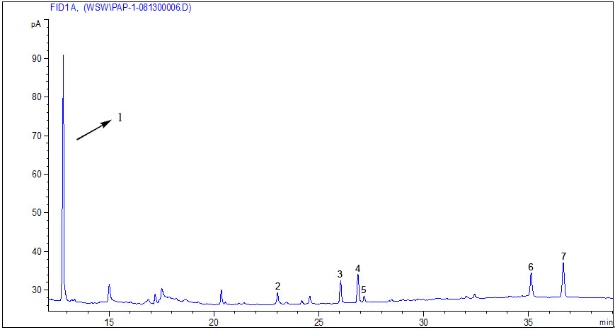

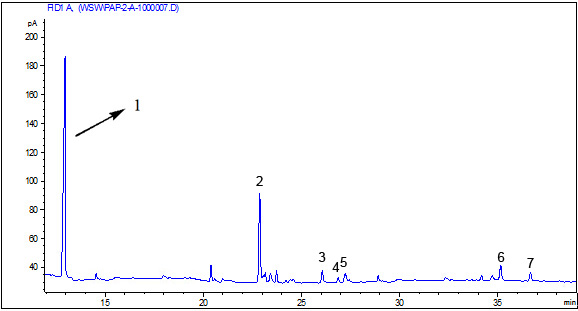


C D

**S-Fig. 8**. GC graphs of standard monosaccharide (A) and Smith degradation products of PRP0 (B), PRP1(C), and PRP2 (D).

| 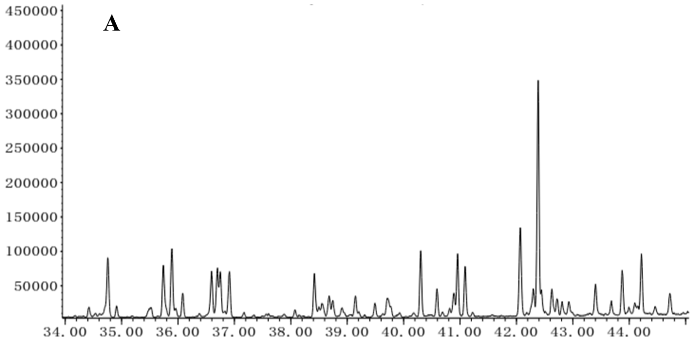 | 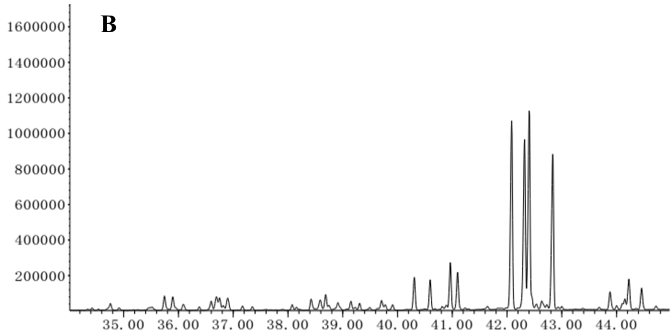 |
| --- | --- |

**S-Fig. 9**. Total ionic chromatograms of methylated PRP1 (A) and PRP2 (B).


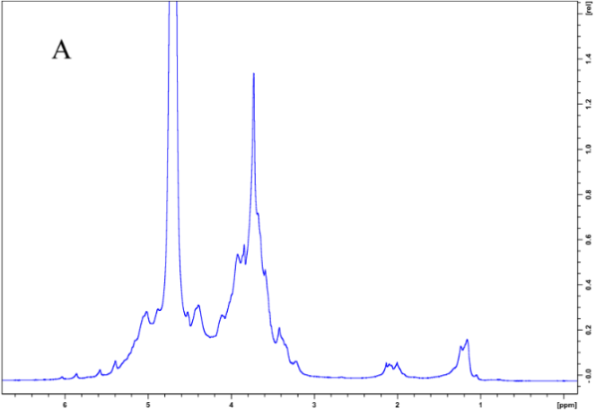

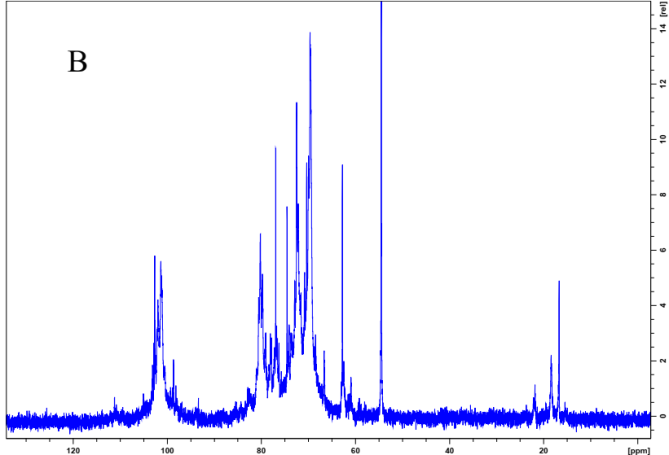


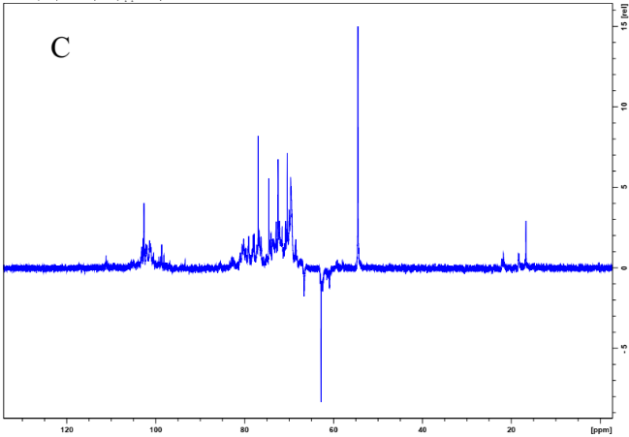

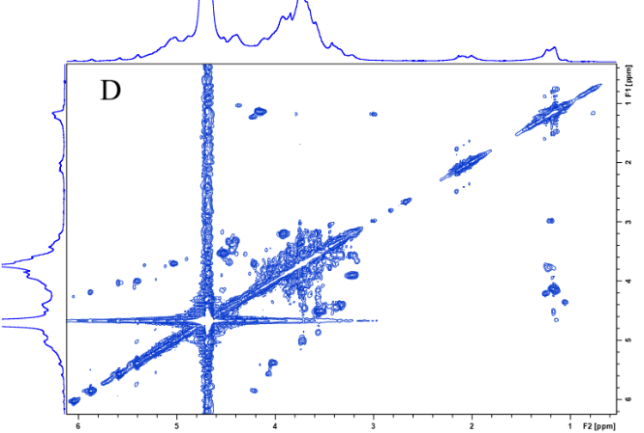


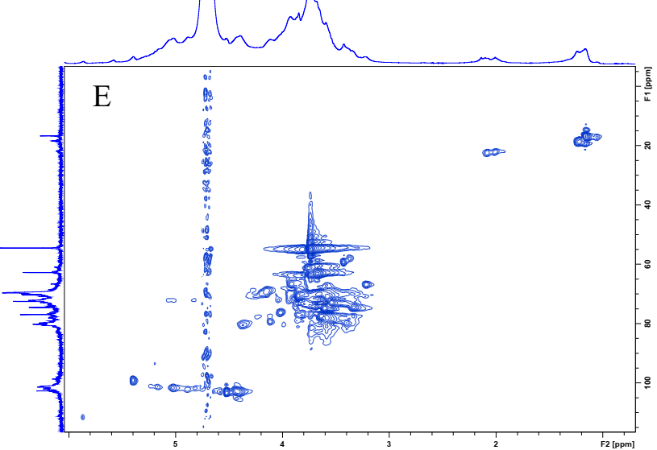

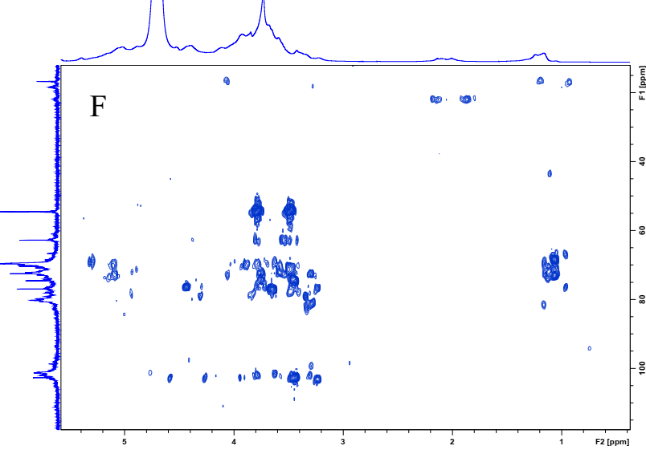


S-Fig. 10. 1D and 2D NMR spectra of PRP1.

(A: ^1^H NMR, B: ^13^C NMR, C: DEPT-135, D: ^1^H-^1^H COSY, E: HSQC, and F: HMBC)


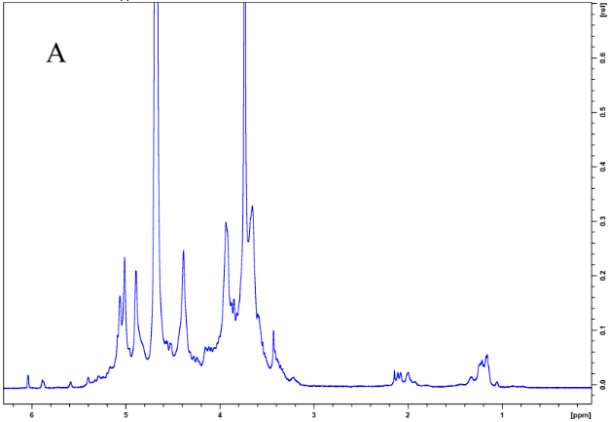

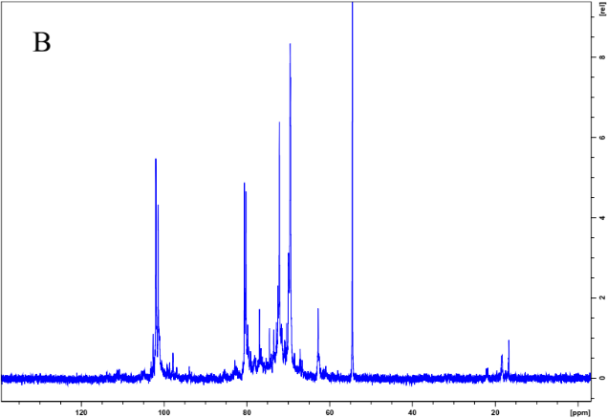


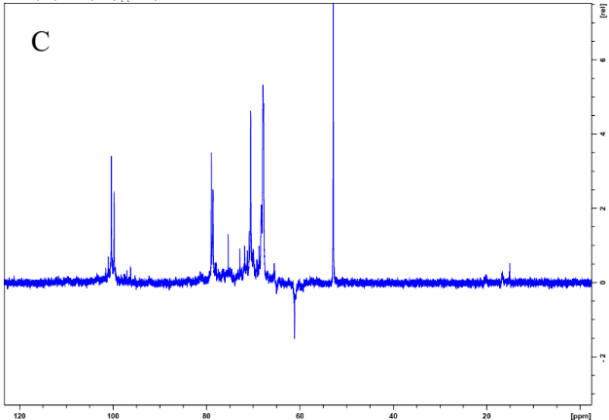

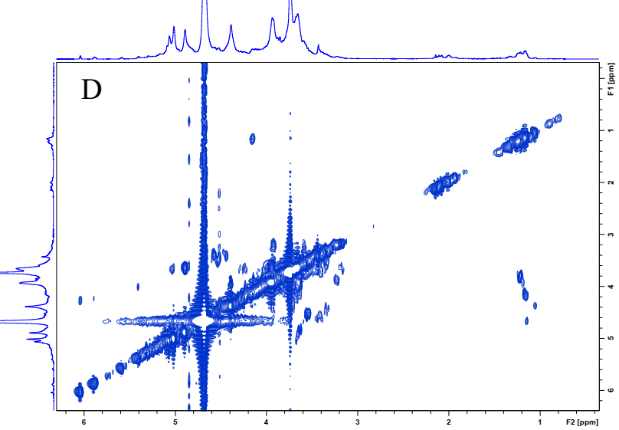


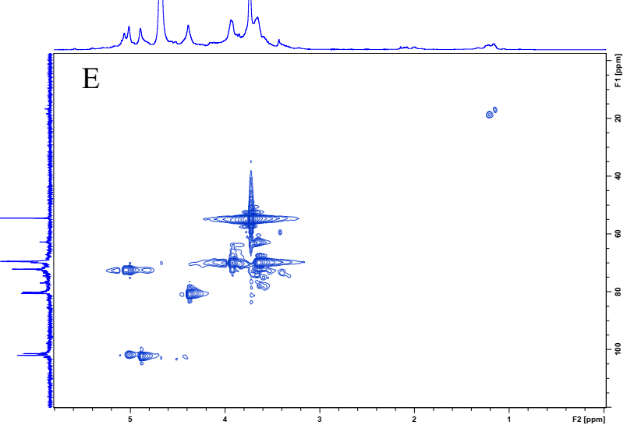

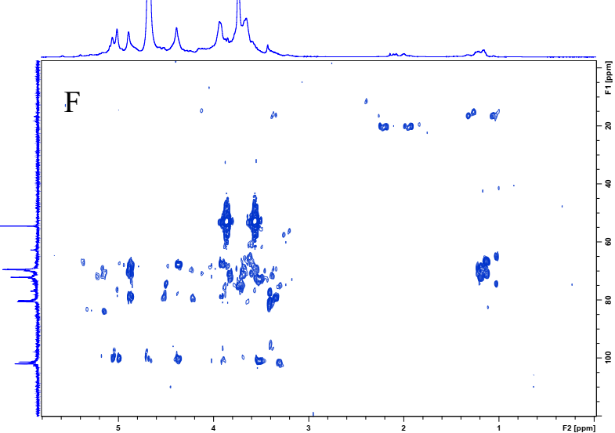


S-Fig. 11. 1D and 2D NMR spectra of PRP2.

(A: ^1^H NMR, B: ^13^C NMR, C: DEPT-135, D: ^1^H-^1^H COSY, E: HSQC, and F: HMBC)

**S-Table 1**. Results of periodic acid in oxidation of homogeneous polysaccharides

| Sample | Reaction Time  (h) | Amount of substance  (mmol) | periodic acid (mol/mol) | HCOOH  (mol/ mol) |
| --- | --- | --- | --- | --- |
| PRP0 | 120 | 0.1265 | 0.935 | 0.400 |
| PRP1 | 120 | 0.1259 | 1.012 | 0.364 |
| PRP2 | 120 | 0.1230 | 0.805 | 0.238 |

**S-Table 2**. Results of products of Smith degradation of homogeneous polysaccharides

| Sample | Gly | Ery | Rha | Ara | Fuc | Xyl | Man | Glc | Gal |
| --- | --- | --- | --- | --- | --- | --- | --- | --- | --- |
| Standard (*R*_t_/min) | 12.85 | 23.28 | 26.17 | 27.13 | 27.38 | 27.61 | 35.29 | 35.69 | 36.81 |
| PRP0 | + | + | + | - | + | - | + | + | + |
| PRP1 | + | + | + | - | + | - | + | - | + |
| PRP2 | + | + | + | - | - | - | + | - | + |

Gly: Glycerol, Ery: Erythritol, +: detected, -: no detected
